# Florid Cemento-Osseous Dysplasia Presenting as Secondary Osteomyelitis: A Case Report of an Undiagnosed Condition

**DOI:** 10.7759/cureus.70291

**Published:** 2024-09-26

**Authors:** Yajas Kumar, Rakshak Anand, Nitin Bhagat, Kapila Chakarvarty, Yashmi Jaiswal

**Affiliations:** 1 Department of Oral and Maxillofacial Surgery, Manav Rachna Dental College, Faridabad, IND; 2 Oral and Maxillofacial Surgery, Private Practice, New Delhi, IND

**Keywords:** cemento-osseous dysplasia, extraction of teeth, fibro-osseous lesions, florid cemento-osseous dysplasia, mandibular osteomyelitis

## Abstract

Florid cemento-osseous dysplasia is a rare, benign fibro-osseous lesion predominantly affecting middle-aged women which is characterized by the presence of multiple radiolucent and radiopaque lesions in the jaw. When complicated by secondary conditions such as osteomyelitis, it may present diagnostic challenges. We present a case underscoring the need for heightened awareness and accurate diagnosis of this disorder, particularly when presenting with complications such as osteomyelitis. Early and precise identification is essential for effective management and to reduce the risk of recurrent symptoms.

## Introduction

Fibro-osseous lesions (FOLs) are a classification of bone lesions characterized by the replacement of normal bone with fibrous connective tissue, which may also contain atypical bone or cementum [1,2]. Lichtenstein first documented FOLs of the head and neck in 1938 [3]. Since then, various categories have emerged, reflecting the ongoing debate and lack of consensus regarding diagnostic criteria. The World Health Organization has categorized osseous dysplasia (OD) into four distinct subtypes: periapical osseous dysplasia, typically localized to the anterior mandible; focal cemento-osseous dysplasia (FCOD), which is predominantly found in the posterior regions of the jaws; florid cemento-osseous dysplasia (FLCOD), characterized by involvement of multiple quadrants of the jaws; and familial gigantiform cementoma (FGC) [4,5]. FLCOD was described first in 1976 [6]. The etiology is indeterminate and the pathogenesis entails a reactive process in the alveolar bone of the jaws, wherein normal bone tissue is replaced by a connective matrix. This matrix comprises a poorly cellularized, cementum-like substance and cellular fibrous connective tissue [6,7]. The nomenclature of FLCOD and other bone diseases may be complex in the literature as multiple terminologies exist [8]. After any dental intervention that might occur without thorough planning, the risk of infection increases in patients with cemento-osseous dysplasia even though the rates have decreased over the decades. The guidelines to classify these lesions as infected FLCOD or osteomyelitis are sparse. It is well established that osteomyelitis is an intraosseous inflammatory disorder affecting both the cortical bone and the periosteum, distinguished by recurrent cycles of bone resorption and formation. The diagnosis is often based on the clinical development of the disease along with radiographic evidence whereas in the case of infected cemento-osseous dysplasia the initiation of the disease depends on the access of oral flora to the underlying dysplasia along with secondary inflammation which may be triggered by chronic periodontal disease, pulpal necrosis, and tooth extraction. Symptoms like dull pain or drainage are typically linked to the presence of sclerotic masses in the affected area, which often indicates secondary osteomyelitis. The management of infected FLCOD is difficult due to diminished vascularization and heightened bone density, which predisposes the affected region to necrosis [9,10]. Here, we describe a case of previously undiagnosed FLCOD manifesting as secondary osteomyelitis.

## Case presentation

**Figure 1:**
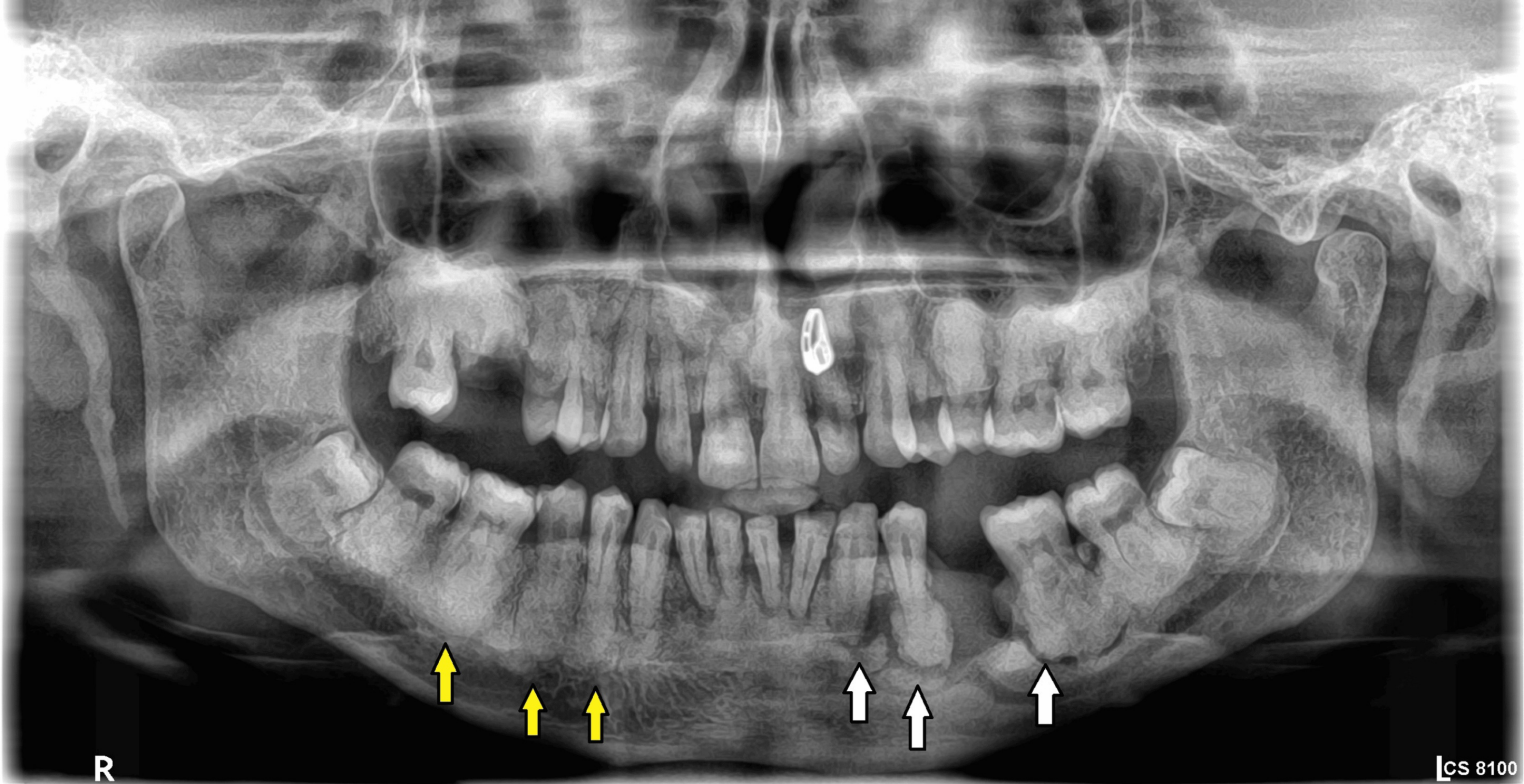
Preoperative panoramic radiograph. Osteolytic lesion present in the region of 34 to 36 can be seen (white arrows) along with dense radio opaque masses throughout the alveolar processes involving the inter radicular bone up to the cementoenamel junction (yellow arrows).

A 31-year-old female reported to the outpatient department of Manav Rachna Dental College, Faridabad, Haryana with a chief complaint of pain and swelling in the left side of the lower jaw. She gave a history of extraction of lower left second premolar from a private practitioner, approximately two years from the date of reporting. after which she experienced dull pain and pus discharge intermittently over the course of the illness and took treatment and opinion from outside. She had no deleterious habits and hematological investigations were within normal limits. The patient had been self-medicating and taking different over-the-counter antibiotics. On examination, the patient had diffuse swelling in the left posterior mandibular region extra orally; grade II mobility with respect to 32, 33, 34 was seen along with vestibular swelling with respect to 36. A grayish-yellow slough was seen with respect to 35 suggestive of impaired healing. Orthopantomogram revealed dense radiopaque masses throughout the alveolar process of upper and lower arches involving the interradicular bone up to the cementoenamel junction with osteolytic lesions present in the region of 34 to 36 (Figure 1).

**Figure 2:**
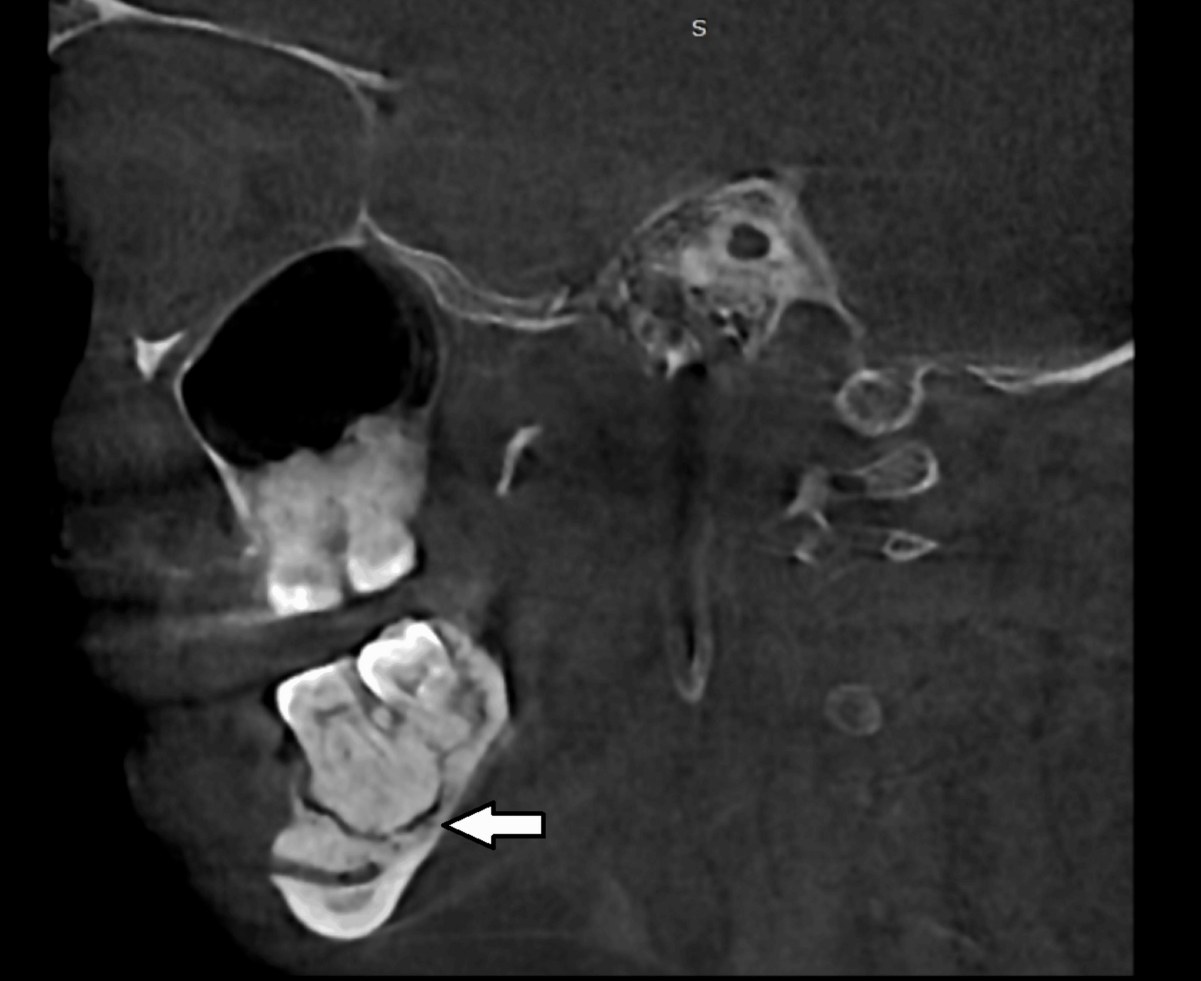
Sagittal section showing the dense radio opaque mass in relation to 36, 37 with osteolytic activity (white arrow).

**Figure 3:**
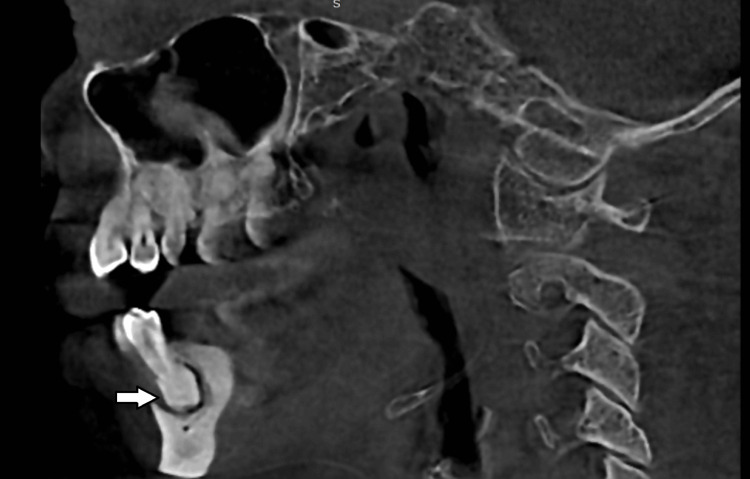
Sagittal section showing the dense radio opaque mass in relation to 34 with osteolytic activity (white arrow).

A cone beam computed tomography scan was obtained for further preoperative evaluation, sagittal section showed the presence of dense radio-opaque mass along with osteolytic activity in 36, 37 region (Figure 2) and in 34 region (Figure 3).

**Figure 4:**
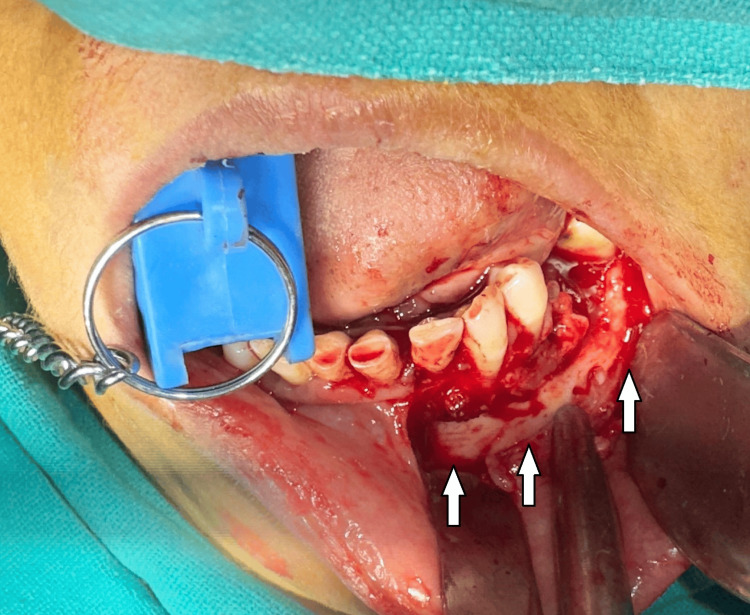
Intraoperative photograph. Raising a full thickness mucoperiosteal flap to expose the sequestrum present with respect to 34, 36, 37 (white arrows).

Biochemical analyses of serum alkaline phosphatase, calcium, and phosphorus were conducted, all of which fell within normal ranges, thereby excluding Paget's disease as a potential diagnosis. After considering all findings, a definitive diagnosis of FLCOD was established, with an associated region of osteomyelitis. All necessary consents were obtained and a plan for sequestrectomy under general anesthesia was made. Under all aseptic conditions, after a successful nasal endotracheal intubation patient was painted with 5% povidone-iodine and draped. Local anesthesia was administered in the form of 2% lidocaine with adrenaline (1:2,00,000) for hemostasis. A crevicular incision was made intraorally from 31-38 with an anterior releasing incision. Full-thickness mucoperiosteal flaps were reflected to expose the sequestrum present with respect to 34, 36, 37 (Figure 4).

**Figure 5:**
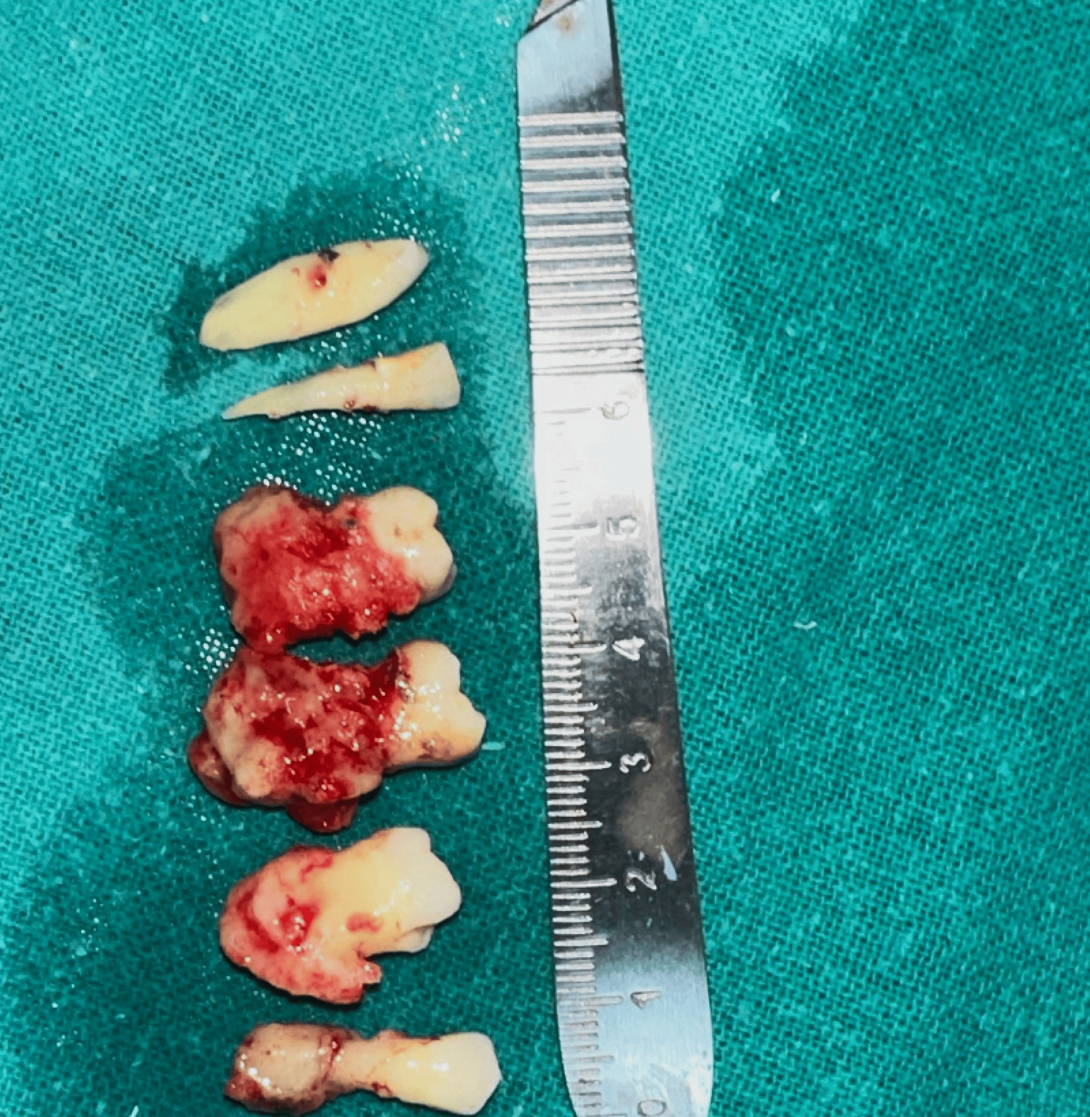
Extracted teeth with associated lesion.

**Figure 6:**
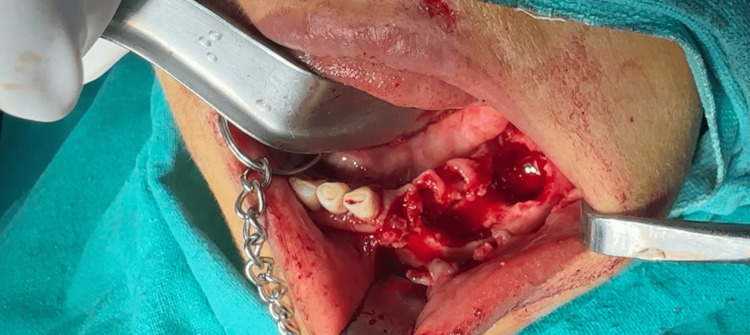
Residual defect with fresh bleeding seen after sequestrectomy and saucerization.

Extraction of 32, 33, 34, 36, 37, 38 (Figure 5) was done carefully followed by sequestrectomy and saucerization using HP No. 8 bur and rose bur (SS White, Lakewood, NJ, USA) under copious irrigation with 0.9% normal saline until fresh bleeding was encountered (Figure 6).

**Figure 7:**
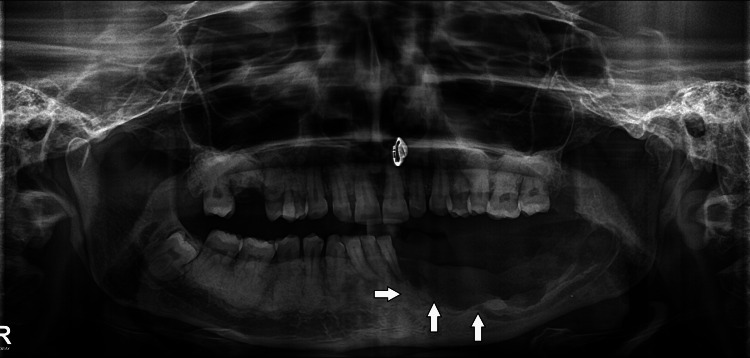
Postoperative panoramic radiograph of one-year follow-up. No signs of osteolytic activity with adequate bone remodelling were seen (white arrows).

The mucoperiosteal flaps were closed primarily with continuous locking sutures using 3-0 silk on a reverse cutting needle (Mersilk, Ethicon, Somerville, NJ, USA). One year post operative radiograph showed adequate bone remodeling without any signs of osteolytic activity (Figure 7).

Patient is now on close follow-up since one year; the wound is healing adequately and there are no signs of paresthesia.

## Discussion

Mesenchymal stem cells appear to have a compromised ability to maintain structural integrity, which leads to the formation of "dysplastic bone" in cemento-osseous dysplasias. However, these cells are neither cancerous nor precursors to cancer [11]. Instead, these cells produce morphologically similar but defective cementum or bone, making the term "dysmorphic bone" more appropriate. This unusual behavior of mesenchymal stem cells underscores the complexity of cemento-osseous dysplasia and raises questions about its underlying mechanisms and potential triggers. Although these lesions are usually asymptomatic and do not require treatment, they can complicate dental imaging and diagnosis, often leading to misinterpretation as more serious conditions. Additionally, understanding the morphological features of these dysplastic formations is essential for developing effective management strategies, especially in distinguishing them from other fibro-osseous lesions that might present complications or require intervention [12]. FLCOD is a benign fibro-osseous lesion primarily affecting middle-aged women, especially those of African descent. Research indicates that the prevalence of FLCOD is significantly higher in this demographic, with studies showing that it is most commonly diagnosed in women aged between 30 and 50 years. This condition is generally recognized as a relatively common occurrence among African American women, although exact prevalence rates can vary by region and population [13].

The hallmark characteristics of FLCOD encompass the occurrence of multiple radiolucent and radiopaque lesions within the jaw. These complex radiographic manifestations can sometimes lead to erroneous diagnoses if not meticulously recognized. While FLCOD is benign and typically asymptomatic, understanding its prevalence and demographic associations is crucial for dental professionals. This awareness can help avoid unnecessary interventions and manage potential complications, such as secondary chronic osteomyelitis [14,15]. Radiographic imaging plays a pivotal role in this differentiation, revealing the characteristic progression of lesions from radiolucent to mixed and finally radiopaque stages, which can guide treatment decisions. Furthermore, timely intervention through debridement and appropriate antibiotic therapy not only alleviates symptoms but also minimizes the risk of further complications associated with untreated infections [16]. FLCOD typically presents as multifocal lesions in the jaws, often detected incidentally on radiographs. Radiographs reveal large radiolucent, mixed, or more commonly, dense radiopaque masses confined to the periapical alveolar bone. These masses do not affect the basilar bone except through direct focal extension and do not appear in the rami. Unlike periapical cemento-osseous dysplasia, these lesions are not always restricted to the periapical alveolar bone; they often extend into the interradicular bone up to the cementoenamel junction. Typically, these lesions do not cause bone expansion, though rare cases may exhibit mild expansion [17]. Symptomatic cases of FLCOD with secondary infection can be managed with local wound care along with antibiotic therapy. For persistent cases, adding hyperbaric oxygen to the treatment plan often improves outcomes by boosting the effectiveness of both the body's immune response and antibiotics in areas with poor blood circulation. While the affected bone-cementum complex generally does not fully heal, these treatments often relieve symptoms and exacerbations commonly recur. In such instances, resolution may be achieved through surgery, specifically an alveolar resection of the symptomatic area [18].

Furthermore, as antimicrobial resistance becomes an increasing concern, particularly with strains such as methicillin-resistant* Staphylococcus aureus*, tailored therapy based on culture and sensitivity results is vital to effectively combat these infections while minimizing side effects and preserving future treatment options. Thus, understanding the interplay between FLCOD and its complications emphasizes the importance of vigilance and adaptability in clinical practice [19,20]. Additionally, the psychosocial impact of FLCOD and its complications should not be overlooked, as patients may experience anxiety or distress due to chronic pain and the uncertainty surrounding their diagnosis. This psychological burden can influence treatment adherence and overall quality of life, making it imperative for healthcare providers to address these concerns through supportive counseling and education [18]. It is worth noting that emerging research into the genetic predispositions associated with FLCOD could provide insights into patient management strategies, potentially leading to personalized approaches that consider individual risk factors for secondary infections [19].

## Conclusions

As our understanding of cemento-osseous dysplasia evolves, integrating such knowledge into clinical practice will enhance the framework for monitoring and treating affected individuals, ultimately developing a more holistic approach to care that encompasses both physical and mental health dimensions. By prioritizing a comprehensive care model, we can empower patients to actively participate in their treatment plans, thereby improving outcomes and promoting resilience in the face of challenges associated with their condition. It is imperative for the healthcare practitioners to thoroughly evaluate each patient before beginning any surgical procedure as the outcomes may be complicated by underlying abnormalities which may go unnoticed at the first instance. It is also essential for proper guidelines and protocols to be in place regarding the management of fibro osseous lesions.
